# Cases of Hernia

**Published:** 1827-11

**Authors:** H. Earle

**Affiliations:** St. Bartholomew's Hospital


### HERNIA.

*Cases of Hernia, treated at* St. Bartholomew's Hospital; *with Observations.* By H. Earle, Esq. F.R.S. &c.

Case I.—John Harris, ætat. fifty-one, a watch-maker by trade, was admitted into St. Bartholomew's Hospital on Thursday night, October 4th, at twelve, with a large strangulated scrotal hernia. It appeared from his account that he had been subject to hernia for many years, but, not suffering materially from it, he had not made use of any truss, and the rupture always remained unreduced. On the Wednesday morning before his admission, he was seized with a fit of sneezing and coughing, and felt the tumor suddenly enlarged to a great size, accompanied with severe pain. This took place when he was endeavouring to evacuate his bowels, which had not acted from the Tuesday morning. Finding the pain increase, with tenderness of the whole belly and vomiting, he called in a surgeon, who made many unsuccessful attempts to reduce the hernia, and at last sent him to the hospital at midnight on Thursday. He was put into the warm bath, and the taxis slightly employed. I saw him between two and three, and immediately resolved to operate. The following was his state at that time:—The tumor was of great size, and very tense; the testicle could be felt at the lower part, but could not be separated from the tumor, which led to the supposition that the hernia was scroto-vaginal. The hernia was very tender to the touch. The abdomen was swollen and very tender, particularly near the ring, which was much distended. His pulse was frequent and very feeble; countenance anxious and sunken; skin clammy; and he had been constantly vomiting for many hours. The bowels had not been relieved from the Tuesday preceding.

On making a long incision through the tumor, the sac, which was much thickened, was found closely adhering to the surface of the intestine, which proved to be the cœcum, with part of the colon. The intestine was much thickened, but not inflamed. On examining the abdominal ring, it was found very large, and free from stricture, the intestine adhered closely all round the inferior margin. Air could be pressed from the tumor upwards in the direction of the ascending colon, and, when the bulk of the tumor was thus a little reduced, a firm elastic-feeling body could be distinguished within the cavity of the sac. This was evidently not a collection of fæces, as it could not be moved. It was obvious that the symptoms of strangulation which existed were not caused by any confinement at the ring, and an accurate examination and careful dissection proved that there was no additional protrusion of small intestine. This circumstance, coupled with the foreign body which was felt within the cœcum, led me to suppose that a portion of small intestine had been protruded through the valve of the colon, forming a case of introsusception within the hernia. With a view to further reduce the tension of the tumor, I punctured the gut with a small trocar, and drew off a small quantity of dirty-coloured fluid and flatus; still the great bulk of the swelling remained but little diminished. The circumstances of the case were very embarrassing, the symptoms urgent, the patient in imminent danger. Nothing presented itself to my consideration but to freely open the bowel which it was impossible to reduce, in consequence of its firm adhesions to the surrounding mass, the nature of which could not be ascertained. The large surface of bowel which had been necessarily exposed, and deprived of all integumental support, rendered it highly probable, even should the passage through the small intestines be restored, that the functions of the cœcum and colon would be suspended, as the intestine would be left to its own spontaneous and insulated efforts to expel its contents, under circumstances most unfavourable, from the adhesions which bound it down on all sides but the front, from the pressure it sustained from the surrounding diseased mass, and from the inflammation which might be expected to follow the operation. From these circumstances alone, an artificial anus was the only alternative which offered; added to which, it appeared evident that the obstruction was connected with the elastic body, which could be felt within the cœcum, in the direction of the valve of the colon. Having proceeded thus far without removing or detecting the cause of the obstruction, it became necessary to investigate further, and, should it prove to be a case of introsusception, to endeavour, if possible, to return the volvulus through the valve. The passage of the ileum behind the cœcum was rendered impossible, from circumstances which became apparent on dissection: to attempt any thing, therefore, with the upper portion of gut, with a view to extricate it from the valve, was quite impracticable. On maturely considering all these circumstances, and entertaining a full sense of the heavy responsibility which a surgeon incurs in resolving to establish an artificial anus, I determined to make an opening at the lower part, immediately opposite to the most prominent part of the projecting internal tumor.

An incision of an inch and a half long was made through the gut: the coats were greatly thickened, but not inflamed. On cutting through the peritoneal and muscular coats, the mucous coat protruded through the incision to a large extent. On opening this, and introducing my finger, I was able to trace the continuity of the gut upwards through the ring, in the course of the ascending colon. The cavity of the tumor was filled with numerous folds of thickened mucous membrane, to which I could not find any opening, except at one spot where a circular opening could be felt, but so small as to lead me to suppose it to be the entrance to the processus vermiformis. As far as I could judge, the opinion I had formed was confirmed by this further examination. The gentlemen present examined the interior of the gut, and came to the same conclusion.

The patient was now so much exhausted, and the case presented so hopeless an aspect, that I did not consider it right to persevere in any attempts to restore the introsuscepted gut. He was accordingly removed to bed, with a large mass of intestine exposed, having an opening at the lower part, through which some flatus passed. His pulse was exceedingly feeble, countenance pallid, and his extremities cold.

An injection of warm water was ordered to be given; the tumor to be fomented with soft wash-leather. Warm port-wine to be taken frequently.—℞. Tr. Opii m. xx.

The following is the daily report of his progress:

October 5th, eight A.M.—Slight reaction has taken place; he has not vomited since the operation; complains of a good deal of pain about the abdomen; nothing but flatus has escaped through the wound; he is much oppressed prior to its passing.

Ten P.M.—Vital powers much depressed; pulse exceedingly low; no fæces have yet passed through the wound.—He continues to take warm wine and sago.

October 6th, eight A.M.—During the night, a considerable quantity of feculent matter has passed through the opening in the gut, through which a portion protrudes, like a prolapsus of the anus; he is not entirely free from pain, which he attributes to the accumulation of flatus. Upon removing the wash-leather which covered the tumor, a discharge of healthy fæces took place. A piece of oil-silk was directed to be placed around the base of the tumor, so as to defend the patient's thighs from the discharge which is continually oozing out, to his great relief.

Ordered arrow-root, sago, and strong broth, ad lib.; with Oj. Vin. rub. per diem.—Tr. Opii p. r. n.

7th.—He states that he feels much relieved; abdomen perfectly soft; he is quite free from pain. A layer of lymph covers the protruded intestine, which in part is becoming organised. Soft feculent matter continues to be discharged through the artificial anus.

Same treatment.

8th.—He continues much in the same state as yesterday. The whole surface of the tumor, excepting a spot about the size of halfa-crown at its lower part, is covered with healthy granulations. His chief complaint is, that he can get no rest at night; his tongue is rather furred; pulse not indicative of much constitutional irritation; he is free from pain.

Tinct. Opii m. xxx.; Aq. M. pip. ℥iss. o. n.—Same diet.

9th.—Much the same as last report.

10th.—The tumor still continues undiminished in size: its lower part, which it was apprehended would slough, is now covered with granulations; feculent matter, of a good yellow colour, is frequently discharged from the wound. Patient much oppressed from the accumulation of flatus, which produces slight griping pains. He has had some difficulty in voiding his urine, which is high coloured. The leather seemed to irritate the granulating surface of the gut.

Let simple dressing be used in its stead, over which place a piece of warm flannel.

11th.—Not quite so well; feels exceedingly low; tongue furred, and complains of slight headache. The opening in the gut remains perfectly free.

Same treatment.

12th.—Has had a restless night; feels much exhausted. The sister states that during the night he was at times delirious. He does not complain of pain, yet his countenance is expressive of great anxiety.

13th.—Much worse; he was again delirious last night; tongue covered with a whitish-brown fur; natural warmth of the body much reduced; pulse eighty, and tremulous.

In addition to the wine, &c. he was ordered ℥iv. brandy per diem, with eggs ad libitum.

October 14th.—Great depression of vital energy; his features are contracted; pulse exceedingly weak and irregular; tongue covered with a brown fur; he has hiccough, subsultus tendinum, and his extremities are cold; in short, he has all the symptoms of approaching dissolution. No feculent matter has passed through the artificial anus since yesterday morning. Some warm water was directed to be injected in the interior of the gut, by means of a hydrocele bottle with elastic tube affixed, with the view of removing any hardened fæces that might be accumulated there; nothing, however, escaped. Bottles of warm water to be applied to his feet.

15th.—He is still alive. During the night he was much convulsed; his whole body is covered with a cold perspiration; no pulse could be felt at the wrist. He expired at seven o'clock in the evening.

16th.—*Dissection of the body, sixteen hours after death.*—On opening the abdomen, there was an appearance of very slight peritoneal inflammation on the surface of the intestines principally, at the points of contact of the different convolutions. The abdominal ring was large, and free from any stricture. The ascending colon and the lower part of the ileum were seen passing through it. The ileum was prevented from descending further by a firm old band, which connected it with the peritoneal surface of the bladder. It (the ileum) was rather contracted near the neck of the sac, but readily dilated on pressing in the finger. After removing the integuments, an incision was made into the tumor, towards the septum scroti, and a quantity of sloughy cellular membrane and pus was exposed. The bulk of the tumor on this side was composed of thickened and diseased cellular membrane, which enveloped the spermatic cord and the portion of ileum which was tightly bound down in contact with the surface of the colon. This mass of diseased cellular membrane was above four inches in length, and three in depth. The portion of ileum from the inner edge of the ring to its termination in the colon, was about seven inches in length; on the outer side of the exposed gut, towards the thigh, there was another mass of condensed cellular membrane and fat, five inches in length, and above three inches and a half in depth. Behind the gut there was a considerable cavity, containing sloughy cellular membrane and pus. The gut itself, which was exposed in the operation, namely, the cœcum and part of the ascending colon, was inseparably connected with these masses of diseased cellular membrane, which kept it stretched and flattened. On slitting up the gut, on that side which had a peritoneal covering, from the part where it had been opened, large folds of thickened mucous membrane were exposed to view, which were much elevated, and the whole presented a convex surface, instead of any concavity: this appearance was produced by the pressure of the tumor on the scrotal side. The valve of the colon was much thickened and prolonged, conveying the idea of a prolapsed anus. This was the portion which had protruded, during life, through the opening which had been made, which allowed of a ready exit for the contents of the small intestines: immediately below this there was a deep cul de sac, into which the finger had passed after the operation. From the situation and prominence of the valve of the colon, it was apparent that this must have been the aperture which was, at the time of the operation, so small, from the swollen mucous membrane, that I mistook it for the opening of the processus vermiformis. The valve of the colon was so much prolonged and so altered in character, that, even when the whole cœcum and colon were opened, some doubts were entertained whether there was not an introsusception of the ileum. The testicle was situated at the bottom of the tumor; its tunica vaginalis, which was inseparably connected with the hernial sac, was opened at its upper part. The testicle was of its natural size, and healthy. The tunica vaginalis was larger than natural, and had contained a small quantity of fluid, which escaped during operation. The whole tumor, when detached from its connexions, was twenty-five inches in circumference, the greater part of which was composed of diseased cellular membrane, behind and around that part of the colon and cœcum which was not covered by peritoneum. The remaining portion of ileum was healthy, as were all the contents of the abdomen.

On maturely considering all the circumstances of this difficult and truly perplexing case, much interesting matter presents itself for deliberation. In the first place, to what cause was the symptoms of strangulation to be referred? The history given by the patient was obviously erroneous, and affords another instance, in addition to very many which I have met with, of the impossibility of placing the slightest reliance on the accounts furnished by patients of the existing, compared with the previous state, of the hernia. That no sudden increase of volume could have taken place, dissection satisfactorily proved: the situation in which he particularly stated this increase to have occurred, namely, towards the septum scroti, was the principal seat of inflammation and sloughing in the thickened cellular membrane, and it is probable that, from some cause, inflammatory action was set up in this part, which, as I shall presently endeavour to explain, was the cause of the obstruction. That this inflammatory action existed before the operation, is rendered almost certain by the pain which he always expressed as greatest in this part; and, from its not having been disturbed or at all denuded of its natural coverings by the operation, it is not likely to have occurred subsequently to the operation. That the symptoms of strangulation were not referrible to mere constipation, may be inferred from their yielding after the operation, without the exhibition of any medicine except opium, and further from the great tenderness which existed in the whole tumor before the operation. To what then, it may be asked, are we to refer the symptoms of strangulation, and their subsequent removal? To form any adequate idea of this, requires that the parts should have been accurately inspected, and I fear that words alone will be insufficient to convey any satisfactory explanation.

It is clear that the hernia must have existed very many years; it is equally so that no sudden increase in the portion of protruded gut could have taken place; and that no difficulty to the transmission of fæces existed at the ring, is equally obvious. To what, then, are we to refer the symptoms? The following explanation I venture to offer as the impression on my mind:—The valve of the colon was much prolonged into the cavity of the cœcum, and its aperture was diminished in consequence of the thickening of its parietes. The cœcum and colon were so much extended in consequence of their firm attachments to the diseased cellular membrane and its great volume, that the natural cavity of these viscera was nearly obliterated; the under surface of the bowel being pressed up, so as greatly to diminish the calibre of the gut, and to keep the thickened mucous surfaces nearly in contact. Such being the state of the gut, a very little increased pressure would be sufficient to compress the sides of the elongated valve together, and to form a complete stoppage to the passage of the contents of the small intestines. Inflammation from some cause appears to have taken place in that part of the tumor which pressed most on the ileum and the valve; and thus, I conceive, a mechanical obstacle was afforded to the passage of fæces.

The relief to all the urgent symptoms of vomiting and tension, which followed the operation, and the subsequent free passage of feculent matter, fully justifies this explanation.

It may, perhaps, be a question in the minds of some, whether I was warranted in making the opening into the cœcum? The cessation of all the more urgent symptoms, and the complete establishment of an artificial anus, might be urged in support of the measure,—if success ought ever to be brought forward in justification of a wrong, or even a doubtful measure. But I do not wish to make my stand on such grounds, as I humbly conceive that the principle on which I acted was justifiable; and I am willing, therefore, to court discussion on the subject, as likely to be beneficial, in the possible occurrence of any similar case.

The urgent symptoms of strangulation which existed when the patient was admitted into the hospital, and his repeated declaration that it had suddenly increased in volume during the exertion of sneezing and coughing, imperiously called for an operation. Having commenced it, and exposed a large surface of intestine, and not having found any recently protruded portion, or any sufficient cause to explain the nature of the constipation, it became necessary to investigate more narrowly that portion which was exposed, and this led to the supposition of an introsusception through the valve of the colon; a supposition which was strengthened by the examination after the opening had been made, and which was not wholly disproved by the complete exposure of the parts after death.

Supposing such an introsusception to have existed, the opening of the bowel, and the establishment of an artificial anus, was the only plan of treatment which afforded even the remotest shadow of a successful issue, under all the circumstances which have been explained in the account of the operation. To have left the patient without making this further effort would not have been justifiable, and would have been a total abandonment of the case. The adoption of this measure unquestionably relieved the more pressing symptoms, and prolonged the patient's existence.

The features presented by this case were altogether new. The situation in which I was placed was difficult and perplexing. The motives which influenced my practice I have endeavoured to explain: at the same time, I wish to express my desire for information from those who may have met with any similar cases, and my openness to conviction should any reasonable objections be raised to the line of conduct which I pursued.

Case II. – Catherine Ann Darn,* ætat. sixty-seven, was brought into the hospital at two o'clock p.m. on Wednesday, the 10th of October, with strangulated femoral hernia. She had been subject to hernia for some years, but had not worn any truss. Some weeks before her admission, the hernia came down, and was reduced with difficulty; the impression being left on the mind of the surgeon that a portion of adherent omentum remained in the sac. The hernia had been strangulated from the previous Sunday, from which day she had been constantly vomiting, and had passed no stools. She had taken purgative medicines without avail, and on the Wednesday applied to the gentleman who had previously relieved her. The symptoms were so urgent at that time, that he immediately removed her to the hospital, without making any attempt at reduction. Her countenance was remarkably sunken; tongue dry, skin clammy; the whole abdomen and tumor very tender to the touch; in a word, nothing but an immediate operation afforded a prospect of benefit. On cutting down, the fascia propria was very distinct and transparent; on opening which, a body, much resembling diseased omentum, presented itself. On dividing cautiously through this, it proved to be adipose substance, much altered in structure, and a small gland in a state of suppuration. The peritoneal sac was closely in contact with a knuckle of much discoloured intestine, the surface of which was partially coated with lymph; not a drop of fluid was contained within the sac. The stricture, which was very close, was situated at the posterior border of the crural arch, commonly called Gimbernat's ligament. On dividing this with the hernial knife, the gut was readily reduced; and, on following it with my finger, I ascertained that it was quite free within the cavity of the abdomen. As the stomach continued irritable after the operation, I directed her to take Tinct. Opii m. xx.; to have injections of warm water; and, if no stools passed in the course of a few hours, to take minute doses of Sulphate of Magnesia in mint-water. Forty leeches were ordered to be applied over the surface of the abdomen.

She took two doses of the Sulphate of Magnesia, when her bowels were satisfactorily moved, and she discontinued it. At eleven p.m. her pulse being hard, and abdomen still tender, she was bled to the extent of twelve ounces.

On the 11th, the bleeding was repeated to the same amount, and twenty more leeches were applied to the abdomen, which was much softer, but still tender to the touch. An enema with Oleum Ricini was thrown up.

From this time she continued to go on very satisfactorily. On the 13th, the stools were frothy and light coloured, and Pulv. Hydrarg. cum Cretâ gr. iv. were ordered to be taken night and morning. The wound in the integuments healed in part by the first intention, but the diseased adipose membrane covering the sac being in a sloughy state, and the gland having suppurated, it was necessary to separate the adhesions, and poultice the wound. At the present time, (October 20th,) the wound is granulating very kindly; the abdomen is soft and free from pain, and she is in every respect in a most favourable state.

The principal points of interest in this case were the successful termination of femoral hernia after four days' strangulation, and the absence of fluid in the sac after so long an interval.

***George-street*; *October 20th,* 1827.**

